# Experimental and Numerical Investigation on the Perforation Resistance of Double-Layered Metal Shield under High-Velocity Impact of Armor-Piercing Projectiles

**DOI:** 10.3390/ma14030626

**Published:** 2021-01-29

**Authors:** Riccardo Scazzosi, Marco Giglio, Andrea Manes

**Affiliations:** Politecnico di Milano, Department of Mechanical Engineering, via la Masa, 1, 20156 Milan, Italy; riccardo.scazzosi@polimi.it (R.S.); marco.giglio@polimi.it (M.G.)

**Keywords:** terminal ballistics, high-velocity impact, aluminum, high-strength steel, double-layer, experimental tests, numerical model, finite element method

## Abstract

In the case of protection of transportation systems, the optimization of the shield is of practical interest to reduce the weight of such components and thus increase the payload or reduce the fuel consumption. As far as metal shields are concerned, some investigations based on numerical simulations showed that a multi-layered configuration made of layers of different metals could be a promising solution to reduce the weight of the shield. However, only a few experimental studies on this subject are available. The aim of this study is therefore to discuss whether or not a monolithic shield can be substituted by a double-layered configuration manufactured from two different metals and if such a configuration can guarantee the same perforation resistance at a lower weight. In order to answer this question, the performance of a ballistic shield constituted of a layer of high-strength steel and a layer of an aluminum alloy impacted by an armor piercing projectile was investigated in experimental tests. Furthermore, an axisymmetric finite element model was developed. The effect of the strain rate hardening parameter C and the thermal softening parameter m of the Johnson–Cook constitutive model was investigated. The numerical model was used to understand the perforation process and the energy dissipation mechanism inside the target. It was found that if the high-strength steel plate is used as a front layer, the specific ballistic energy increases by 54% with respect to the monolithic high-strength steel plate. On the other hand, the specific ballistic energy decreases if the aluminum plate is used as the front layer.

## 1. Introduction

The function of a ballistic shield is protection against external threats. The architecture, and more specifically the thickness of the shield, is designed according to the required protection criteria. In the case of protection from projectile impact, the ballistic shield thickness depends on the penetration capability of the projectile which is in turn mainly determined by its hardness and kinetic energy. In order to stop armor-piercing (AP) projectiles, particularly hard materials are necessary, which are usually either high-strength steel or ceramics. In the case of protection of vehicles, the weight of the shield is of concern and the aim is to increase the payload while keeping the fuel consumption low. Therefore, the optimization of the shield, which is the minimization of the mass of the shield required to withstand a specified threat, is of practical interest. Steels are characterized by high strength and hardness combined with high ductility and a low price, and, compared to more sophisticated armor materials, have excellent load carrying capability and formability [1].

As far as metal shields are concerned, several studies focused on the perforation resistance of multi-layered metal shields. Ben-Dor et al. already presented two reviews in 2012 [2] and 2017 [3] concerning analytical, numerical and experimental investigations about the effect of layering, spacing and change of the order of plates. Experimental and numerical studies which focused on the effect of layering and the order of layers of different metals are resumed in Table 1.

Dey et al. [4] found that for a blunt projectile, layering increases the ballistic limit velocity by 50% for plates in direct contact and by 40% for plates with an air gap. For ogival projectiles, layering decreases the ballistic limit velocity by 10% for both plates in direct contact and with an air gap. Borvik et al. [1] found that the ballistic limit velocity is not affected by layering if the plates are in contact while it clearly decreased if the two plates were separated by a 30 mm air gap. Flores-Johnson et al. [5] numerically investigated the performance of several multi-layered configurations using Weldox 700E and Al7075-T651 plates. They found that Al7075-T651 6.66 mm + Weldox 700E 13.33 mm showed the highest perforation resistance. This finding showed that the ballistic resistance of armor shields can be potentially increased by using a multi-layered configuration with different materials. Babaei et al. [6] studied the perforation resistance of several double-layered targets using 1 mm steel or aluminum plates, finding the highest ballistic resistance with 1 mm steel + 1 mm steel configuration. They also found that 1 mm steel + 1 mm aluminum configuration performs better than 1 mm aluminum + 1 mm steel configuration.

In 2012 Ben-Dor et al. [2] reviewed the research about the ballistic performance of metal multi-layered shields and the main conclusions, stated by the authors as “cautious”, were: (i) most often the ballistic resistance is decreased by layering and is further degraded by spacing; (ii) the ballistic performance of spaced shields is lower if the number of layers is higher and is affected by the order of layers of different thickness; (iii) ballistic performance may strongly depend on the order of layers of different materials; (iv) the ballistic performance is less affected by layering at higher impact velocities and (v) the character and the magnitude of the effect of layering and spacing depends on the geometry of the projectile and the target.

Yunfei et al. [7] investigated the perforation resistance of double-layered targets constituted by plates of two steels of different strength: 45 steel, which has a yield strength of 714 MPa, and Q235 steel, which has a yield strength of 299 MPa. It was found that the ballistic limit velocity was higher by 7.2% for the blunt projectile, and by 2.1% for the ogival projectile, if the plate with higher strength was used as the front layer. Rahman et al. [8] studied with numerical simulations the high-velocity impact of a 7.62 mm armor piercing projectile against multi-layered targets constituted by high-strength steel and Al7075-T6 plates. The target configuration high-strength steel 8 mm + Al7075-T6 9 mm + high-strength steel 8 mm was found to be an interesting solution since it led to weight reduction while improving the perforation resistance of the shield.

In 2017 Ben-Dor et al. [3] presented a second review which included new investigations and a few earlier studies that were not included in the first review [2]. Using information from both the reviews the authors stated that the effect of layering and spacing cannot be predicted and only general trends can be observed. In some circumstances, layering and spacing influence is small, comparable to the magnitude of experimental errors, and thus the results are not very reliable. The general patterns in the case of pointed projectiles are: (i) the ballistic performance is degraded by layering and spacing; (ii) the ballistic performance decreases if the number of spaced layers increases and (iii) is strongly influenced by the order of plates of different materials. In the case of blunt projectiles: (i) in most cases layering and spacing decreases the ballistic performance but the influence is lower with respect to ogival projectiles and (ii) the ballistic performance depends on the order of layers of different materials or of different thickness.

Holmen et al. [9] investigated the perforation resistance of hot-rolled structural steel plates both in the as-received and case-hardened state. Case hardening increased the ballistic limit velocity by at least 20% while layering reduced the ballistic performance of the case-hardened plates more than the as-received plates. This is potentially due to the fact that as-received plates show more global plastic deformation as the number of layers increases, counteracting the expected decrease in perforation resistance, while this does not happen for case-hardened plates, which show almost no global deformation. Zahari et al. [10] analyzed nine different double layered configurations by means of finite element analysis which consisted of plates of the same 3 mm thickness made of different materials: steel, aluminum or titanium. The best configuration in terms of specific energy absorption was given by the configuration titanium 3 mm + aluminum 3 mm. Rahman et al. [11] studied by means of numerical models the performance of triple-layered targets consisting of plates of steel Ar500 and AA7075-T6 using different joining materials: non-joining material, epoxy, polyurethane or Al-Si-Zn filler metal. The configuration with polyurethane as joining material was shown to give the best performance. Rahman et al. [12] studied two target configurations: 15 mm Ar500 steel + 10 mm AA7075-T6 and 10 mm AA7075-T6 + 15 mm Ar500 steel. The projectile was a 7.62 mm full metal jacket bullet with a lead core. No complete perforation was obtained at impact velocities ranging between 800–850 m/s. The penetration depth was six times higher in the target configuration with AA7075-T6 as the front layer.

The finite element method with Lagrangian approach is the traditional choice for developing a numerical model of high-velocity impact on metal shields. The numerical model is usually three-dimensional [5,13,14,15,16,17,18,19,20,21,22,23] but also an axisymmetric model [1,4,24] has been used in the literature. The former is able to simulate more realistically the physical characteristics of the phenomenon but requires far more computational resources. In terminal ballistics, the Johnson–Cook model [25] is the most popular constitutive relation used to predict the mechanical behavior of metals under high-velocity impact [1,4,5,13,14,15,16,18,19,20,21,22,23,24,26,27]. This constitutive relation takes into account the effect of plastic strain hardening, strain rate hardening and thermal softening. Strain rate hardening of metals is evaluated by performing mechanical tests at different levels of strain rate. This result is achieved using the Split Hopkinson Pressure Bar testing apparatus [28,29,30]. Several failure criteria were used for metals. The Johnson–Cook failure criterion [31] expresses the failure strain as a function of stress triaxiality, strain rate and temperature. The effect of stress triaxiality can be assessed by performing tensile tests with notched specimens with different curvatures. The strain rate effect is evaluated by performing Split Hopkinson Pressure Bar tests while the temperature effect is evaluated by performing tensile tests at different temperatures [29]. For metals, fracture strain increases with increasing temperature while it decreases with increasing stress triaxiality and strain rate [31]. The Cockcroft–Latham failure criterion [32] assumes that the failure is controlled by the integral of the maximum principal stress over the equivalent plastic strain. The main advantage of the Cockcroft–Latham failure criterion is that it is defined by only one material constant which can be evaluated by a tensile test. Furthermore, it is able to capture the behavior for most steels exposed to impact [1]. Bai and Wierzbicki [33] postulated a failure criterion for metals which considers both pressure sensitivity and the Lode angle dependence. Gilioli et al. [34] applied this failure criterion to simulate high-velocity impact on AA6061-T6 aluminum alloy.

Numerical studies [5,8,10] showed that it is possible to decrease the weight of the shield using layers of different metals. Among the published papers, only [6,12] experimentally investigated the shields with layers of different metals (steel and aluminum alloy), but no comparison was made with a monolithic shield of the same weight. Only one of these studies actually measured the ballistic limit velocity [6], but the investigation was based on thin plates and blunt projectiles. To the authors’ knowledge, an experimental investigation using the commercial ammunition 7.62 mm armor-piercing projectile, and target thicknesses closer to the common application, is still missing. The aim of this study is therefore to investigate using experimental evidence if a monolithic shield can be substituted by a double-layered configuration manufactured from two different metals while guaranteeing the same perforation resistance at a lower weight. According to the authors opinion this question has not been clearly answered in the available literature and an experimental investigation of this topic is necessary. The authors already presented a similar study [35], but using a commercial ammunition 7.62 mm with a soft-core. The behavior of a soft-core projectile is completely different from the behavior of an armor-piercing projectile [1]. For this reason, different results were obtained, as described in the text. Therefore, this study aims at investigating both experimentally and with numerical models the performance of a ballistic shield composed of a layer of high-strength steel and a layer of an aluminum alloy impacted by a 7.62 mm armor-piercing projectile. The effect of the order of the two layers was evaluated to decide which of the two materials to use as the front layer and the performance of either of these two double-layered shields was compared with a steel monolithic plate with similar areal density. Furthermore, the ballistic curve and the ballistic limit velocity was determined for each configuration. The numerical models, based on the finite element method, allowed a better comprehension of the problem. In Section 2 the experimental results are reported: normal impact tests at different velocities were conducted to determine the ballistic curve and the ballistic limit velocity of each shield. The development of the numerical model is reported in Section 3: axisymmetric finite elements models were developed, and the target material input parameters were investigated simulating impact on monolithic targets. In Section 4 the accuracy of the numerical model is assessed by comparison with experimental results for double-layered targets. The numerical models developed were subsequently used to understand the energy dissipation mechanism inside the target.

## 2. Experimental Tests

Three different targets were subjected to a high-velocity impact to evaluate their performance in terms of ballistic limit velocity as summarized in Table 2. Target S was a monolithic 6.94 mm thick Ramor 500 steel plate. The other two targets were two double-layered shields with similar areal density composed of two plates: a 3.23 mm thick Ramor 500 steel plate and an 8.27 mm thick AA6061-T6 aluminum plate. In target SA the Ramor 500 plate was the front layer. On the other hand, in target AS the AA6061-T6 plate was the front layer. The thickness of the two layers was chosen according to the availability of the supplier, with the aim being to get an areal density similar to the monolithic plate. The two plates were kept in contact by the clamping system of the experimental apparatus with no air gap. The projectile was a 7.62 × 51 P80: the diameter was 7.8 mm, the mass was 9.75 g and the nominal impact velocity was 820 m/s. It was an armor piercing projectile with a hardened steel core, a brass jacket and a lead end, equivalent to the FB7 protection level of the standard EN 1522 [36]. According to the manufacturer specifications, 14.5 mm of Ramor 500 are required to obtain the FB7 protection level. Thus, complete perforation was expected for target S at the projectile nominal impact velocity. The targets had dimensions of 500 × 500 mm and were impacted nine times with different impact velocities to obtain the ballistic curve. The in-plane distance between each shot and the boundary was larger than 100 mm in order to assure no reciprocal influence in the results [1]. The impact and the residual velocity of the projectile was measured by two speed traps which were positioned 2.5 m in front and behind the target.

Ballistic curves were fitted, by means of the method of least squares, through the experimental points with the Lambert–Jonas equation [37] defined in Equation (1)

(1) $$v_{r}=a{\left(v_{i}^{p}-v_{bl}^{p}\right)}^{\frac{1}{p}}$$

where *v_i_* and *v_r_* are respectively the projectile impact and residual velocity, *v_bl_* is the ballistic limit velocity and *p* are two empirical constants. The ballistic limit velocity was therefore calculated as equal to the parameter *v_bl_* of the fitted Lambert–Jonas equation. The specific ballistic energy was finally computed as the ratio between the kinetic energy of the projectile at the ballistic limit velocity and the areal density of the target. Thereby the performance of target S, as well as targets SA and AS, which had slightly different areal density, could be compared. The experimentally measured impact and residual velocities are reported in Table 3.

**Figure 1:**
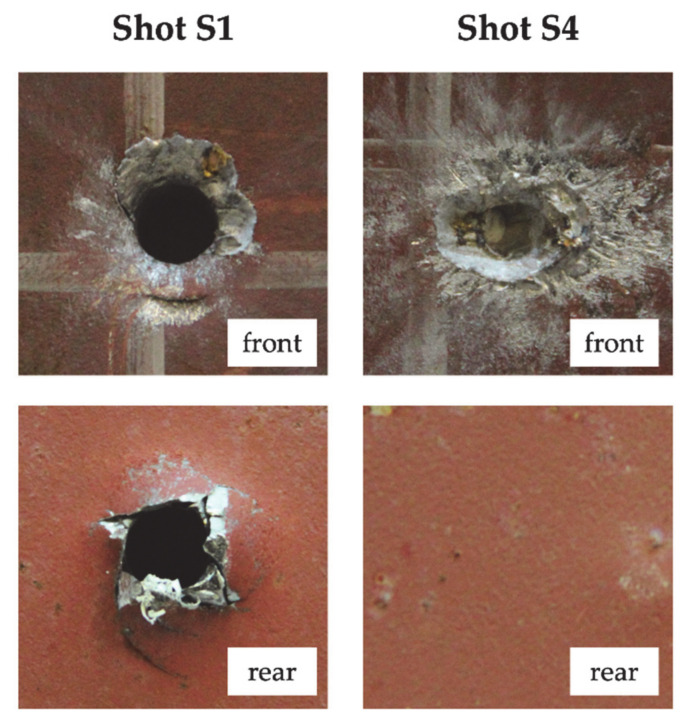
Damage morphology at nominal impact velocity (shot S1) and projectile arrest (shot S4).

The computed ballistic limit velocity of target S was 534.75 m/s. The plate failed by ductile hole radial expansion with neither significant plastic deformation nor bulging in the rear face (see Figure 1). Except for shot S8 (impact velocity of 900 m/s), the hole generated by the projectile had a diameter in the range 5.54–5.84 mm, slightly smaller than the projectile core diameter of 6 mm. For an impact velocity of 900 m/s the hole was consistently larger, with a diameter of 9.06 mm. In case of no perforation (shots S4 and S6), neither a hole nor deformation was visible on the rear face of the target (see Figure 1).

**Figure 2:**
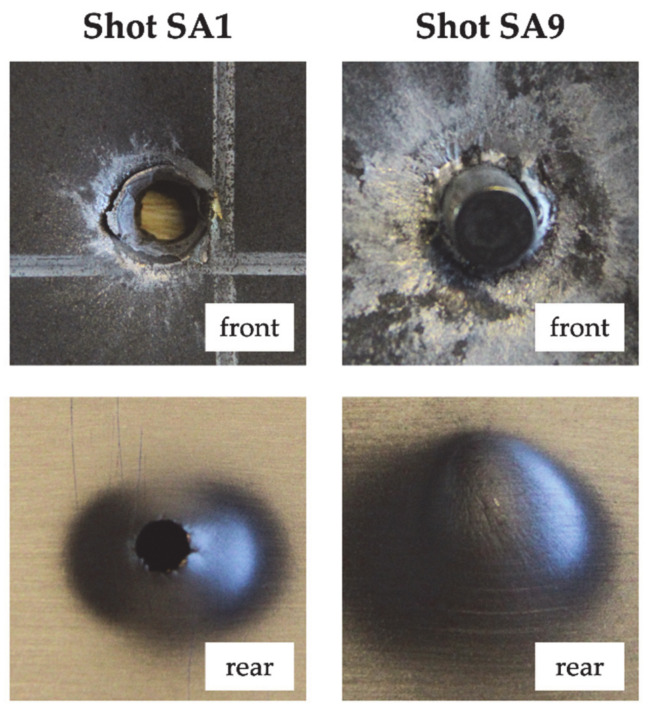
Damage morphology at nominal impact velocity (shot SA1) and projectile arrest (shot SA9).

The computed ballistic limit velocity for target SA was 620.43 m/s. The front steel plate and the rear aluminum plate failed by ductile hole radial expansion with considerable bulging (see Figure 2). For shots SA3, SA5 and SA8 the projectile did not completely perforate the front steel plate. Nevertheless, due to the deformation of the front steel plate the rear aluminum plate showed indentation in the front face and bulging in the rear face. For shot S9 the impact velocity was higher, therefore the projectile completely perforated the front steel plate. It remained stuck in the steel plate but the projectile tip fractured and its fragments only partially perforated the rear aluminum plate. Indeed, as shown in Figure 2, no hole was visible on the rear face of the aluminum plate.

**Figure 3:**
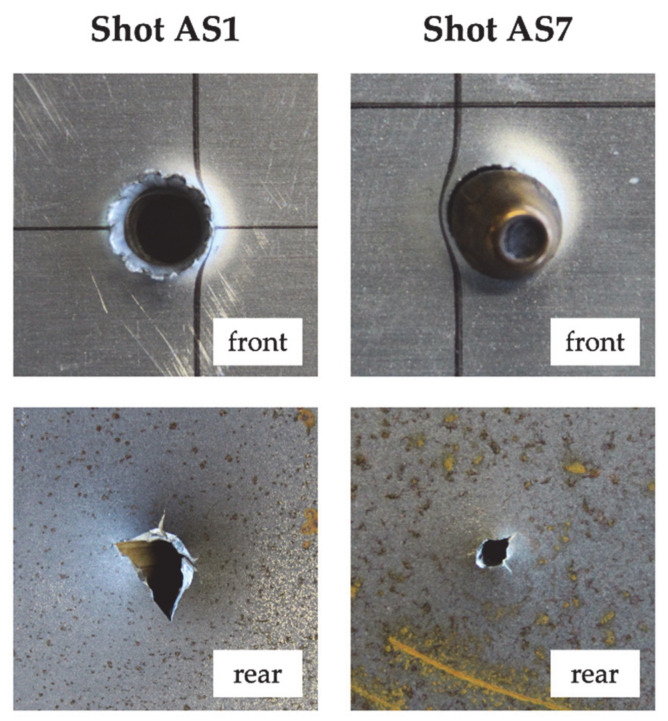
Damage morphology at nominal impact velocity (shot AS1) and projectile arrest (shot AS7).

Regarding target AS, the residual velocity of shot AS8 was not measured due to an error of the speed trap, while the residual velocity of shot AS9 was significantly lower than for other shots at a similar impact velocity, possibly due to inclined impact. For these reasons, shots AS8 and AS9 were not considered in the interpolation of the Lambert–Jonas equation. The computed ballistic limit velocity was 438.54 m/s. The projectile jacket remained stuck in the front aluminum layer which exhibited petaling in the front face and plastic deformation. The rear steel layer showed significant bulging and bending deformation. The hole in the rear steel plate was circular except for shots AS1, AS2 and AS3, which were characterized by the highest impact velocities (between 622.73 and 833.3 m/s), for which the hole exhibited significant fragmentation (see Figure 3). For shots AS5 and AS6 (low measured residual velocity), part of the hardened steel core remained stuck in the front aluminum plate but some fragments were able to perforate the rear steel plate and a residual velocity was measured by the speed trap. For shot AS8, almost all the core remained stuck in the front aluminum plate. A small hole was visible in the rear steel plate (see Figure 3), with a diameter of approximately 2 mm but no moving object was detected by the rear speed trap.

**Figure 4:**
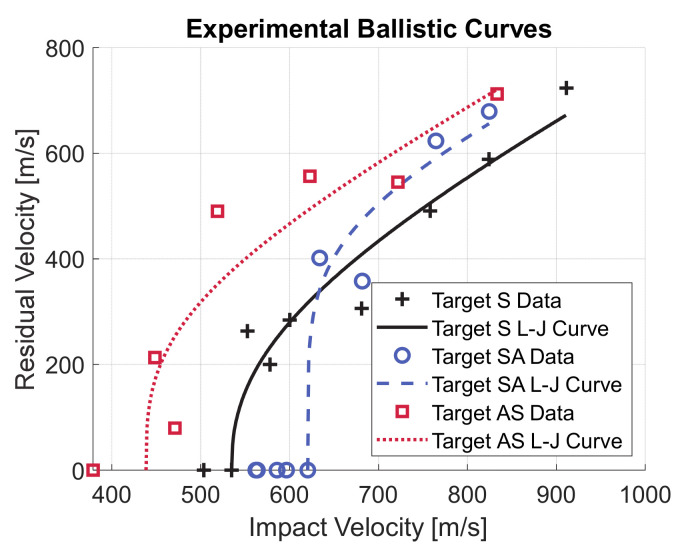
Experimental data and fitted ballistic curves.

**Figure 5:**
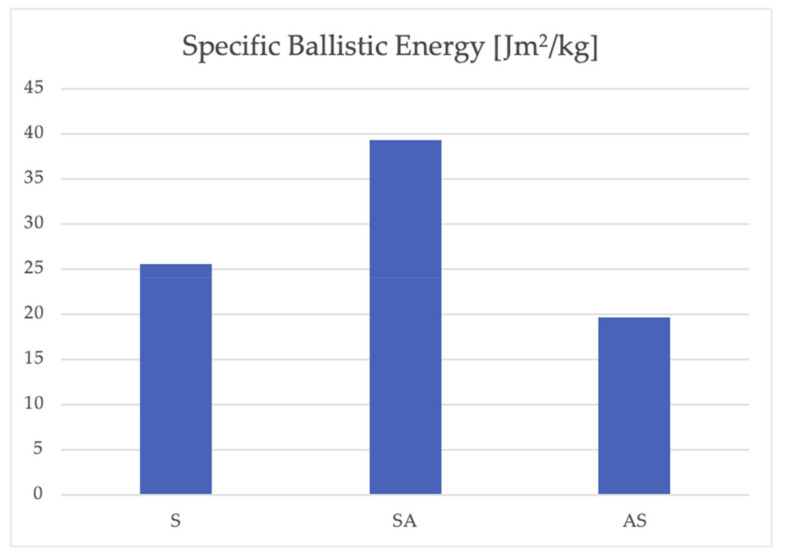
Specific ballistic energy for the three different targets.

The fitted ballistic curves are reported in Figure 4. At the nominal impact velocity, target S shows the best performance since the projectile residual velocity is 588.61 m/s. However, the areal density of target S is slightly higher than the other two targets. Target SA performs slightly better than target AS since for the former the residual velocity is 679.14 m/s while for the latter it is 712.17 m/s. A different observation can be made if the impact velocity is lower, around 600 m/s. Target SA is able to stop the projectile at an impact velocity of 620.43 m/s while target S, despite its higher areal density, is completely perforated at an impact velocity of 600.15 m/s (the residual velocity is 284.39 m/s). Target AS is the worst configuration with a residual velocity of 556.39 m/s for an impact velocity of 622.73 m/s. The specific ballistic energy for the three configurations is shown in Figure 5. Target SA shows considerably higher ballistic limit velocity than target S, while the areal density is slightly lower. Consequently, the specific ballistic energy of target SA is 54% higher than the specific ballistic energy of target S. On the other hand, the specific ballistic energy of target AS is 23% lower than of target S.

Therefore, it was shown that using a double-layer configuration may improve or worsen the ballistic performance. In particular, a configuration with a hard steel plate as a front layer and a soft aluminum plate at the rear layer performs better than a weight equivalent monolithic steel plate. This result is different from what was found by the authors themselves in [38]. In that study a similar double-layered configuration showed lower ballistic performance than the equivalent monolithic shield, when impacted by a soft-core projectile. Thus, the result obtained in this study is limited to the type of projectile used in the experimental tests. However, the increase in the thickness of the shield is a drawback of the increase of performance, since aluminum has a lower density than steel. The order of the different plates plays an important role in the determination of the ballistic performance. In the case studied, the configuration with the aluminum plate as a front layer (target AS) has almost half the specific ballistic energy of the configuration with the steel plate as a front layer (target SA). This finding is in agreement with the experimental results of Babaei et al. [6], Yunfei et al. [7] and Rahman et al. [12], which also observed that better ballistic performance is obtained if the stronger material is placed as the front layer of a double-layered configuration. This is also in agreement with the results obtained by the authors for soft-core projectiles [38]. A possible explanation of the experimental results is that that in the case of target SA, the hard steel plate fractures the projectile tip decreasing its penetration capability. The rear aluminum plate instead has the function of supporting the front layer and absorbing the kinetic energy of the fragments of the projectile (as observed in shot SA9). This projectile defeat mechanism is more efficient than a monolithic plate and similar to the Small Arms Protective Insert (SAPI) plate, the latter being manufactured from a combination of ceramics and ballistic fibers/composites.

## 3. Numerical Model

### 3.1. Numerical Model Development

An axisymmetric finite element model of the high-velocity impact test was developed using the software LS-DYNA R11.1. The material model used for both the target materials (Ramor 500 and AA6061-T6) and the projectile (hardened steel, brass and lead) was MAT_107—Modified Johnson–Cook [39] which is the modified version of the Johnson–Cook constitutive model [25]

(2) $$\sigma_{eq}=\left(A+B\epsilon_{eq}^{n}\right){\left(1+{\dot{\epsilon }}_{eq}^{*}\right)}^{C}\left(1-T^{*m}\right)$$

where *σ_eq_* is the equivalent stress and $\epsilon_{eq}$ is the equivalent plastic strain. *A*, *B* and *n* are material constants which describe the yield stress as a function of equivalent plastic strain at the reference strain rate and room temperature. The material constant *C* is the strain rate hardening parameter and ${\dot{\epsilon }}_{eq}^{*}={\dot{\epsilon }}_{eq}/{\dot{\epsilon }}_{0}$ is the dimensionless plastic strain rate, where ${\dot{\epsilon }}_{0}$ is the reference strain rate. The material constant m is the thermal softening parameter, and the homologous temperature is defined as $T^{*}=\left(T-T_{r}\right)/\left(T_{m}-T_{r}\right)$ where *T* is the absolute temperature, T_r_ is the room temperature and T_m_ is the melting temperature.

The temperature increment due to adiabatic heating is defined as

(3) $$\Delta T=\int_{0}^{\epsilon_{eq}}\chi \frac{\sigma_{eq}d\epsilon_{eq}}{\rho C_{p}}$$

where *χ* is the Taylor–Quinney coefficient, which is a parameter that represents the amount of plastic work converted into heat, *C_p_* is the specific heat and *ρ* is the density.

In this work the Cockcroft–Latham failure criterion was adopted, which is defined as

(4) $$W=\int_{0}^{\epsilon_{eq}}\langle \sigma_{1}\rangle d\epsilon_{eq}\leq W_{c}$$

where *W* is plastic work per unit volume, *σ*_1_ is the maximum principal stress and *W_c_* is the critical value of plastic work per unit volume. Additionally, a temperature based failure criterion was used: when 90% of the melting temperature is reached, the element is removed from the analysis [1,5].

The input parameters related to the projectile were taken from the literature [1,27,29,34,40] and are reported in Table 4. The input parameters related to the target are reported in Table 5. The authors already determined the elastic modulus *E*, the Johnson–Cook parameters *A*, *B* and *n*, and the Cockcroft–Latham parameter *W_c_* either for Ramor 500 [35] or AA66061-T6 [41]. In particular two sets of these parameters were obtained for two plate thicknesses of Ramor 500: around 3 and 6.5 mm. The other material parameters were taken from the literature [1,5,29,34]. More details are given in Section 3.2 regarding the determination of the Johnson–Cook parameters *C* and *m*. In terminal ballistics it is usually assumed that the effect of friction between the projectile and the target is small or even negligible [42]. Thus, the contact between the projectile and the target plates was frictionless. A static and dynamic friction coefficient equal to 0.55 [43] was used for the contact between the steel and aluminum plate in target SA and AS.

**Figure 6:**
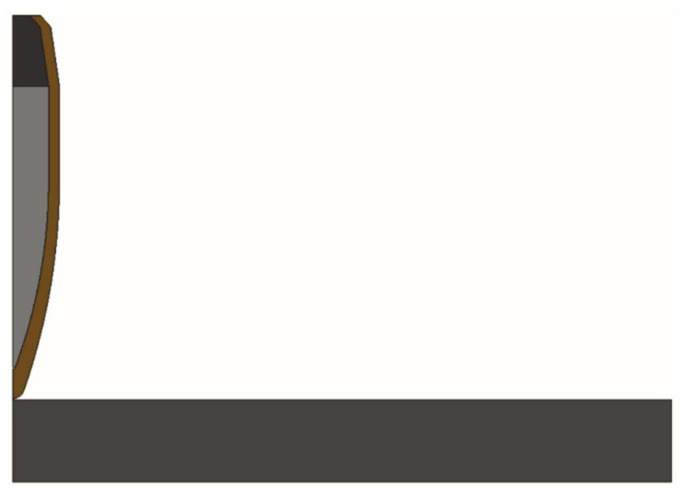
Geometry of the numerical model (target S is shown).

**Figure 7:**
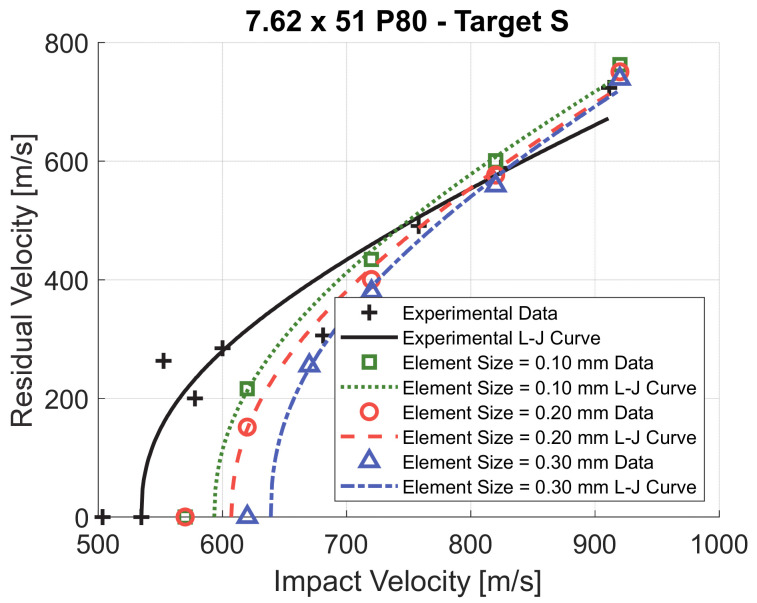
Mesh convergence analysis for the element size of the plate (target S).

The geometry of the numerical model (target S) is shown in Figure 6. Only a portion of the target, corresponding to a radius of 55 mm was modeled and encastre boundary conditions were applied at the outer edge. Axisymmetric shell elements were used for both the projectile and the target. The average mesh size of 0.1 mm was enough to accurately reproduce the geometry of the projectile. The minimum characteristic length was 0.006 mm, defined as the ratio between the element volume and the area of the largest side. This value led to an initial time step of 1.15 × 10^−9^ s. As shown in Figure 7, mesh convergence investigation was carried out on the entire ballistic curve in order to choose the correct mesh for the plate. A mesh size of 0.1 mm was adopted for both target S, SA and AS which was the smallest element size at a reasonable computational cost. This was the element size which gave the best accuracy of the numerical model for target S. The number of elements was 49,604 for target S and 74,940 for target SA and AS. The computational time was around 30 min for target S and 1 h for target SA and AS (using eight processors in shared memory parallel processing mode).

### 3.2. Ramor 500 Material Parameters

**Figure 8:**
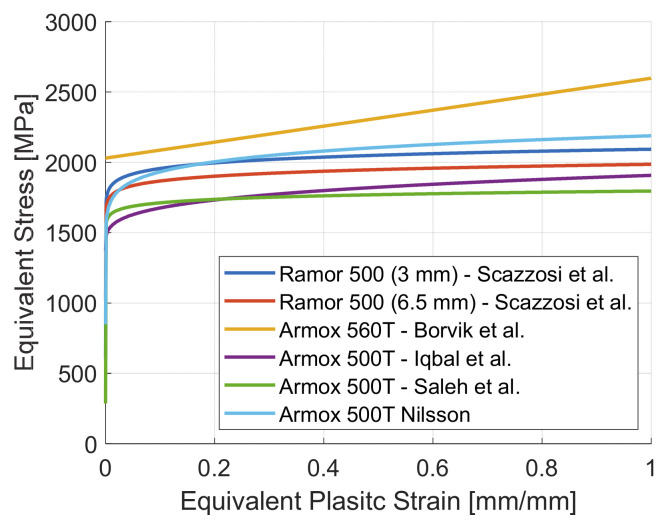
Johnson–Cook static flow stress rule ($\sigma_{eq}=A+B\epsilon_{eq}^{n}$) for different high strength steels.

Ramor 500 is a high-strength steel commonly used in ballistics shields design. According to the manufacturer datasheet, the perforation resistance of Ramor 500 is similar to another steel from the same manufacturer, which is ARMOX 500. Indeed, for both steels, a thickness of 6.5 mm and 14.5 mm is required to obtain respectively a FB6 and FB7 protection level defined in EN 1522 [36]. The authors already determined the elastic modulus *E*, the Johnson–Cook parameters *A*, *B* and *n*, and the Cockcroft–Latham parameter *W_c_* for Ramor 500 [35]. The Johnson–Cook static flow stress rule of Ramor 500 [35] is compared with ARMOX 500T [28,29,44] and ARMOX 560T [1] in Figure 8. Ramor 500 shows a similar static flow rule of ARMOX 500T. Since the authors were unable to identify the Johnson–Cook strain hardening parameter *C* and thermal softening parameter *m* for Ramor 500, they were assumed to be similar to the other high strength steels reported in Figure 8.

**Figure 9:**
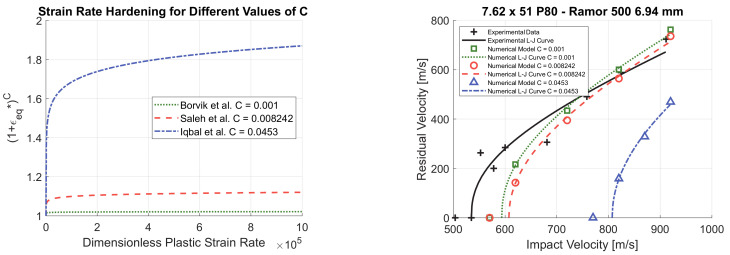
Strain rate hardening for different values of *C* (**a**) and *C* parametric study (*m* = 0.84) (**b**).

As shown in Figure 9a, the values reported in the literature for the parameter *C* vary in a large range, between 0.001 and 0.0453 [1,28,29,44]. Iqbal et al. [29] determined a value of 0.0453. This value is much higher than values obtained by other authors and leads to high strain hardening. As a result, if *C* = 0.0453 is considered, the numerical model considerably overestimates the perforation resistance of target S, as shown in Figure 9b. By decreasing the value of *C*, the perforation resistance predicted by the numerical model decreases and the value of *C* = 0.001, found by Borvik et al. [1], leads to the highest accuracy.

**Figure 10:**
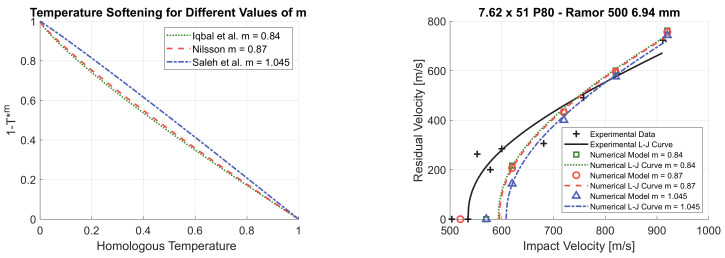
Temperature softening for different values of *m* (**a**) and *m* parametric study (*C* = 0.001) (**b**).

The values reported in the literature for the parameter *m* vary in the range between 0.84 and 1.045 [1,28,29,44]. As shown in Figure 10a, the thermal softening remains largely unaffected if the parameter *m* remains within this range. Considering the maximum and the minimum value of *m*, the maximum difference in the thermal softening rule ($1-T^{m}$) is 12% and is obtained at T* = 0.34. Therefore, the perforation resistance predicted by the numerical model is slightly affected by the value of *m*, as shown in the parametric study of Figure 10b. The perforation resistance slightly decreases if the value of *m* decreases. The highest accuracy is obtained with the value of *m* = 0.84, determined by Iqbal et al. [29].

In conclusion the set of values *C* = 0.001 and *m* = 0.84 were chosen to lead to the highest accuracy of the numerical model. The error of the prediction of the residual velocity is below 5% for impact velocities above 750 m/s. At lower impact velocities the accuracy is worse, and the ballistic limit velocity is overestimated by 11%.

### 3.3. AA6061-T6 Materials Parameters

**Figure 11:**
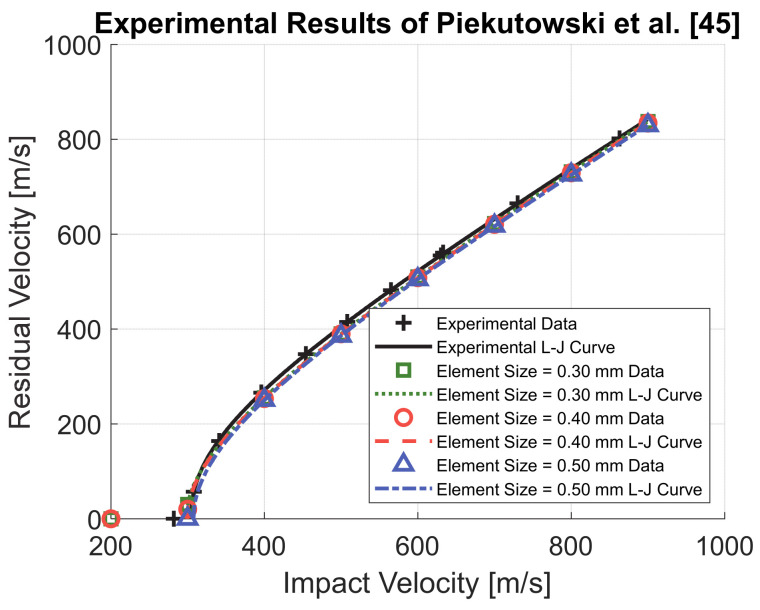
Validation of AA6061-T6 material model and mesh convergence analysis.

The authors already determined the elastic modulus *E*, the Johnson–Cook parameters *A*, *B* and *n*, and the Cockcroft–Latham parameter *W_c_* for AA66061-T6 [41]. The other material parameters were obtained from the literature. Herein, the material model, already used in [41], is further validated by replicating the experimental tests performed by Piekutowski et al. [45] were an ogive-nose steel projectile was impacted against a 26.3 mm thick AA6061-T651 plate. An axisymmetric numerical model which replicates the experiment was built. The projectile had a radius of 12.9 mm (for more details the reader is referred to [45]) and MAT_003—Plastic Kinematic [39] was used. An elastic modulus of 202,000 MPa and a yield strength of 1430 MPa were used, which were reported in [45]. The mesh average size of the projectile was 0.3 mm. The radius of the target was 152 mm and encastre boundary conditions were applied at the outer edge. MAT_107—Modified Johnson–Cook was used [39] with the material parameters reported in Table 5 used for the target. Different mesh element sizes were adopted, ranging from 0.3 to 0.5 mm, to perform a mesh convergence analysis. The results are reported in Figure 11. The numerical model replicated the experimental results with high accuracy and convergence of the results obtained.

## 4. Discussion

### 4.1. Numerical Model Validation

**Figure 12:**
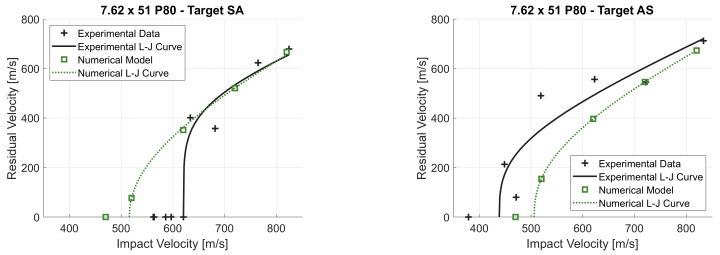
Experimental and numerical ballistic curve for target SA (**a**) and AS (**b**).

The numerical model was validated by simulating the high-velocity impact tests on double-layered targets, maintaining the mesh size of 0.1 mm and the material parameters determined in Section 3. As shown in Figure 12a, the numerical model for target SA is very accurate in the prediction of the residual velocity for impact velocities higher than 625 m/s. The experiential ballistic curve shows a consistent drop in the residual velocity around impact velocity of 625 m/s. Indeed, at 633 m/s the experimental residual velocity was 400 m/s (shot SA6). On the other hand, at 620 m/s the projectile was stopped (shot SA9). The numerical model is unable to capture this sudden change in the residual velocity for very similar impact velocities and therefore underestimates the ballistic limit velocity by 17%. As shown in Figure 12b, the numerical model predicts the same overall shape of the ballistic curve of target AS. The numerical ballistic curve is slightly shifted rightwards, meaning an overestimation of the perforation resistance, but experimental points largely deviated from the experimentally fitted Lambert–Jonas curve, thus it is difficult to assess the model accuracy. Indeed, if the experiential points over 700 m/s impact velocity are considered, the accuracy of the numerical model is high. For example, if shot AS2 is considered (721 m/s impact velocity) the difference between the experimental and numerical residual velocity is negligible. Finally, the ballistic limit velocity is overestimated by 15%.

The authors were unable to find numerical models simulating a high-velocity impact on double-layered targets with different metal alloys which were validated though a direct comparison with experimental tests. Borvik et al. [1] simulated high-velocity impacts on monolithic and double-layered targets with axisymmetric numerical models with errors up to 12% in the estimation of the ballistic limit velocity. Flores-Johnson et al. [5] validated a three-dimensional numerical model simulating high-velocity impacts on monolithic shields, considering two different materials. For the steel Weldox 700E the error on the ballistic limit velocity was 4%, while for the aluminum alloy AA7075-T651 the error was 11%. As reported in Section 3.3, the error in the estimation of the ballistic limit velocity in case of AA6061-T6 was negligible. In the case of target S, as reported in Section 3.2, the error was 11%. The precision of the numerical model for monolithic targets was thus similar to the numerical models of Borvik et al. [1] and Flores-Johnson et al. [5] whereas the inaccuracy of the model for multilayer targets was higher. Two potential explanations might be given for this inaccuracy: (i) experimental tests results, especially for target AS, showed visible deviation from the Lambert–Jonas equation. This deviation cannot be accounted by a numerical model, which perfectly fits the Lambert–Jonas equation. This results in a larger deviation between the experimental and numerical results; (ii) the projectile core which remained stuck in the targets was partially fragmented. The numerical model was uncapable to capture the core fragmentation and this incapability to correctly simulate the fragmentation of the projectile affects the accuracy of the model.

### 4.2. Analysis of the Perforation Process

**Figure 13:**
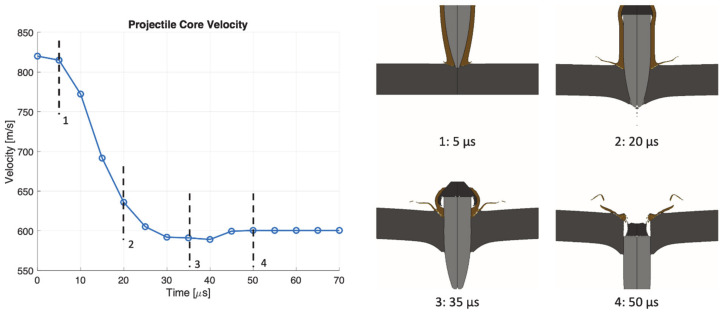
Perforation process in target S at 820 m/s impact velocity.

The perforation process in target S at 820 m/s impact velocity is shown in Figure 13. The lead end and the brass jacket are almost completely destroyed during the impact, while the hardened steel core is only subjected to minimal erosion of the tip. After 20 µs the hole is opened at the rear face of the plate but the projectile core is still subjected to deceleration since it has to enlarge the hole to achieve complete perforation. After 50 µs, the core reaches a constant velocity.

**Figure 14:**
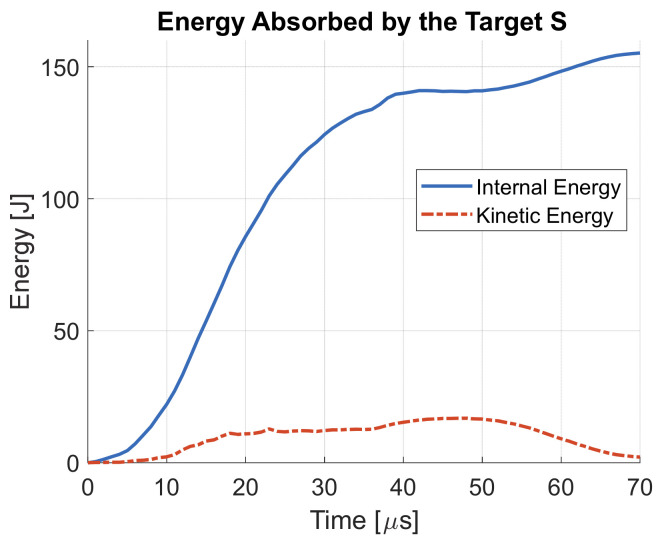
Energy absorbed by the target S at 820 m/s impact velocity.

The energy absorbed by the target S at 820 m/s impact velocity is shown in Figure 14. Since the plate is subjected to limited bending deformation, but only localized plastic deformation up to failure, most of the absorbed energy is converted into internal energy of the target.

**Figure 15:**
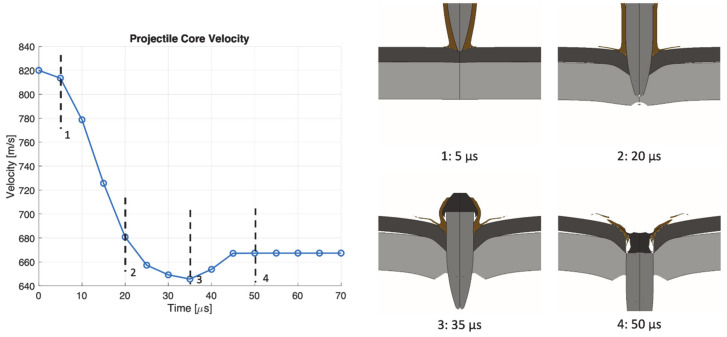
Perforation process in target SA at 820 m/s impact velocity.

**Figure 16:**
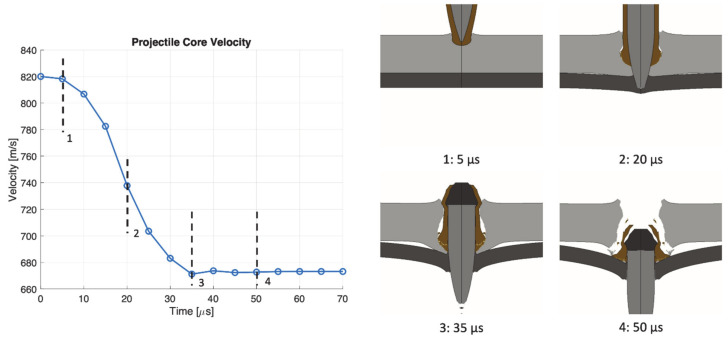
Perforation process in target AS at 820 m/s impact velocity.

The perforation process in target SA at 820 m/s is shown in Figure 15. The projectile core completely perforates the target with minimal erosion on the tip, while the jacket and the end are almost completely destroyed. At 20 µs the aluminum plate begins to fail by tension at the rear face. A constant velocity of the core is reached around 50 µs. The perforation process in target AS at 820 m/s is shown in Figure 16. The projectile core completely perforates the target with minimal erosion on the tip. The jacket also contributes to the perforation of the front aluminum plate, generating a large hole with respect to target SA. The jacket is almost completely destroyed when impacting against the rear steel plate. The projectile reaches a constant velocity after 50 µs.

**Figure 17:**
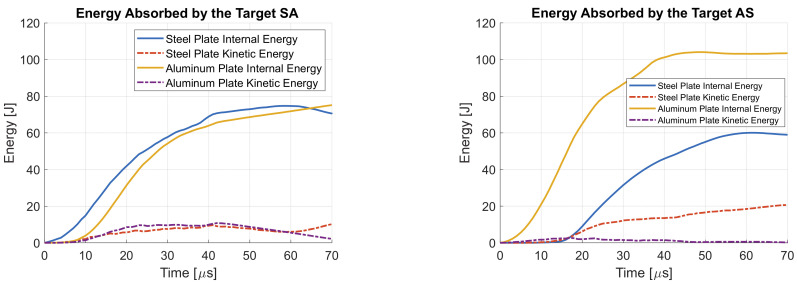
Energy absorbed by the target SA (**a**) and AS (**b**) at 820 m/s impact velocity.

As shown in Figure 17, a different behavior of target SA and AS is reflected in different energy dissipation mechanisms. Target AS is not supported by the aluminum plate, thus it is subjected to large bending deformation. This is reflected in a higher kinetic energy with respect to target SA. On the other hand, in target SA, localized bulging is more pronounced, thus the steel plate internal energy is higher. In target SA the aluminum plate is pushed by the steel plate and it is subsequently subjected to considerable bending deformation. This results in a higher kinetic energy which is comparable to the kinetic energy of the steel plate. On the other hand, in target AS the aluminum plate is not bent by the projectile thus the kinetic energy is lower. In target AS the hole generated in the aluminum plate is larger due to the impact with the jacket, thus its internal energy is higher with respect to target SA. The energy absorbed by target SA and AS at 820 m/s impact velocity is similar, thus the predicted residual velocity is similar. The predicted residual velocity is 667 m/s for target SA and 673 m/s for target AS. This is in agreement with experimental results. The experimental residual velocity was 679 m/s for target SA and 712 m/s for target SA. However, in the case of target AS the impact velocity was 833 m/s while it was 824 m/s for target SA. In the numerical model 820 m/s was kept for both of the two targets, thus a slightly lower difference between the residual velocities must be expected. At 820 m/s impact velocity, the residual velocity for target S predicted by the numerical is 600 m/s, which is 10% lower than target SA. Again, this is in agreement with experimental results. Thus, it can be stated that the accuracy of the numerical model is high at nominal impact velocity. It was experimentally observed that while the specific ballistic energy of target SA is higher than target S, the residual velocity is higher for the former at nominal impact velocity. This means that at lower impact velocities, around 620 m/s, the perforation resistance of SA is higher. This consideration is based on the specific ballistic energy, thus, at first approximation, it does not depend on the weight of the shield. On the other hand, at higher impact velocities, it seems that target S performs better than target SA because the residual velocity is lower. Nevertheless, the residual velocity depends on the weight of the shield. In this sense the performance of target S and SA at nominal impact velocity are not comparable since they have a slightly different areal density. For this reason, a numerical model of high-velocity impact against monolithic plate with the same areal density of target SA is developed. It corresponds to a Ramor 500 plate of 6.07 mm. The predicted residual velocity is 650 m/s, only 3% higher than target SA. Thus, at the same areal density, the monolithic plate performs better than the double-layered target but the difference in the performance is not significant.

## 5. Conclusions

The perforation resistance of double-layered ballistic shields manufactured by plates of different metals were experimentally investigated and compared with the performance of a monolithic target manufactured with high-strength steel of similar areal density. The double-layer shields were constituted of two plates: a Ramor 500 steel plate with a thickness of 3.23 mm and an AA6061-T6 aluminum plate with a thickness of 8.27 mm. Two multi-layer configurations were tested, one in which the steel plate was the front layer and one in which the aluminum plate was the front layer. Ballistic curves were experimentally determined for all the targets, impacted by an armor piercing projectile, and therefore the ballistic limit velocity and the specific ballistic energy were computed. It was experimentally observed that, for the impact conditions considered, using a double-layered configuration may improve or worsen the ballistic performance. In particular, a configuration with a hard steel plate as a front layer and a soft aluminum plate at the rear layer performs better than a weight equivalent monolithic steel plate. The order of plates in different material plays an important role in the determination of the ballistic performance. In the case studied, the configuration with the aluminum plate as a front layer has got almost half the specific ballistic energy of the configuration with the steel plate as a front layer. The effect of the order of the plates is explained by the different behaviors of the individual plates in the two double-layered configurations, as shown by the numerical simulations. An axisymmetric finite element model of the high-velocity impact test was developed. The effect of the strain rate hardening parameter *C* and the thermal softening parameter *m* of the Johnson–Cook constitutive model was investigated. The numerical model was able to predict the ballistic curve with high accuracy in the case of a monolithic target. Lower accuracy was obtained for double-layered targets. The numerical model was exploited to understand the perforation process and the related energy dissipation mechanisms.

## Figures and Tables

**Table 1:** Studies about the effect of layering and the order of layers of different metals.

Authors	Target Configuration	Projectile	Approach
Dey et al. [4]	Weldox 700E 2 × 6 mm	blunt and ogival	experimental
Borvik et al. [1]	Weldox 700E 2 × 6 mm	7.62 mm AP	experimental
Flores-Johnson et al. [5]	various with Weldox 700E and Al7075-T651	7.62 mm AP	numerical
Babaei et al. [6]	steel 1 mm + steel 1 mm steel 1 mm + aluminum 1 mm aluminum 1 mm + steel 1 mm aluminum 1 mm + aluminum 1 mm	blunt	experimental
Yunfei et al. [7]	6 mm 45 steel + 6 mm Q235 steel 6 mm Q235 steel + 6 mm 45 steel	blunt and ogival	experimental
Rahman et al. [8]	various with high-strength steel and Al7075-T6	7.62 mm AP	numerical
Holmen et al. [9]	structural steel 2 × 6 mm structural steel 4 × 3 mm	7.62 mm AP	experimental
Zahari et al. [10]	various with steel, aluminum and titanium	blunt	numerical
Rahman et al. [11]	Ar500 8 mm + Al7075-T6 10 mm + Ar500 7 mm	7.62 mm AP	numerical
Rahman et al. [12]	Ar500 15 mm + AA7075-T6 10 mm AA7075-T6 10 mm+ Ar500 15 mm	7.62 mm lead core	experimental

**Table 2:** High-velocity impact tests target configurations.

Target ID	Target Configuration	Information
S	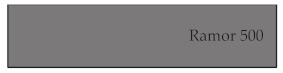	Monolithic Ramor 500 6.94 mm Areal Density: 54.48 kg/m^2^
SA	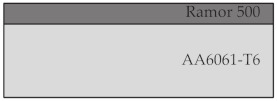	Double-Layered Front Layer: Ramor 500 3.23 mm Rear Layer: AA6061-T6 8.27 mm Areal Density: 47.68 kg/m^2^
AS	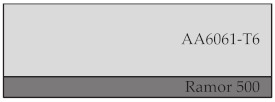	Double-Layered Front Layer: AA6061-T6 8.27 mm Rear Layer: Ramor 500 3.23 mm Areal Density: 47.68 kg/m^2^

**Table 3:** Experimentally measured impact and residual velocity for targets S, SA and AS.

Shot ID	Impact Velocity [m/s]	Residual Velocity [m/s]
Target S: Ramor 500 6.94 mm		
S1	824.22	588.61
S2	681.01	306.08
S3	600.15	284.39
S4	534.75	0
S5	577.93	199.97
S6	503.52	0
S7	758.06	490.82
S8	911.35	723.52
S9	552.59	263.22
Target SA: Ramor 500 3.23 mm + AA6061-T6 8.27 mm		
SA1	824.47	679.14
SA2	681.69	357.81
SA3	585.89	0
SA4	562.03	0
SA5	563.73	0
SA6	633.78	401.69
SA7	764.6	623.39
SA8	596.52	0
SA9	620.43	0
Target AS: AA6061-T6 8.27 mm + Ramor 500 3.23 mm		
AS1	833.3	712.17
AS2	721.83	545.32
AS3	622.73	556.39
AS4	518.9	490.31
AS5	471.05	79.58
AS6	448.52	213.27
AS7	378.91	0
AS8	514.5	not measured
AS9	578.32	101.88

**Table 4:** Projectile materials input parameters [1,27,29,34,40].

Property	Hardened Steel (Core)	Brass (Jacket)	Lead (End)
Density ρ [kg/m^3^]	7850	8520	10,660
Elastic Modulus E [MPa]	200,000	115,000	16,000
Poisson Ratio ν [-]	0.30	0.31	0.42
Specific Heat C_p_ [J/kgK]	455	385	124
Taylor–Quinney Coefficient χ [-]	0.9	0.9	0.9
Expansion Coefficient α [K^−1^]	1.2 × 10^−5^	1.9 × 10^−5^	2.9 × 10^−5^
Reference Strain Rate ${\dot{\epsilon }}_{0}$ [s^−1^]	1	1	72.108
Melting Temperature T_m_ [K]	1800	1189	525
J-C Parameter A [MPa]	1657.71	111.69	0
J-C Parameter B [MPa]	20,855.6	504.69	55.552
J-C Parameter n [-]	0.651	0.42	0.0987
J-C Parameter C [-]	0.007248	0.0085	0.126
J-C Parameter m [-]	0.35	1.68	1
Cockcroft–Latham Parameter W_c_ [MPa]	915	91	175

**Table 5:** Target materials input parameters [1,5,29,34,35,41].

Property	Ramor 500 (3.23 mm)	Ramor 500 (6.94 mm)	AA6061-T6
Density ρ [kg/m^3^]	7850	7850	2700
Elastic Modulus E [MPa]	197,468	201,383	70,216.66
Poisson Ratio ν [-]	0.33	0.33	0.33
Specific Heat C_p_ [J/kgK]	452	452	890
Taylor–Quinney Coefficient χ [-]	0.9	0.9	0.9
Expansion Coefficient α [K^−1^]	1.2 × 10^−5^	1.2 × 10^−5^	2.3 × 10^−5^
Reference Strain Rate ${\dot{\epsilon }}_{0}$ [s^−1^]	5 × 10^−4^	5 × 10^−4^	597.2
Melting Temperature T_m_ [K]	1800	1800	925
J-C Parameter A [MPa]	606.44	1021	198.07
J-C Parameter B [-]	1486.86	965	322.95
J-C Parameter n [-]	0.042	0.057	0.30
J-C Parameter C [-]	0.001	0.001	0.107
J-C Parameter m [-]	0.84	0.84	1.34
Cockcroft–Latham Parameter W_c_ [MPa]	1285	1600	133.10

## Data Availability

Data sharing not applicable.
